# 

**Published:** 1850-06

**Authors:** 


### CONTENTS

### OF NO. VI.

---

### ORIGINAL COMMUNICATIONS.

### ESSAYS AND CASES.

ART. I.—Epidemic Puerperal Peritonitis. By WM. KENNEY, M. D., of Millersburg, Ky. - - - - - 462
ART. II.—A Sudden and Fatal Case of Purpura. By NAPOLEON B. ANDERSON, M. D., of Louisville, Ky. - 470
ART. III.—A Case of Extensive Fracture of the Skull and Rupture of the Meninges, with loss of the Brain, and Subsequent Recovery. By JOHN SWAIN, M. D., of Ballardsville, Ky. - - - - - - - - 473
ART. IV.—The London Lancet for April, on Fractures, Hemorrhage, and Gangrenous Ulcers. By THEODORE S. BELL, M. D., Louisville, Ky. - - - - - 477

### REVIEWS.

ART. V.—On the Treatment of Fractures, &c., by the use of the Roller. By B. W. DUDLEY, M. D., Professor of Surgery in Transylvania University. Transylvania Medical Journal. 1850. Vol. 1, No. v. - - - - 508
ART. VI.—The Diseases of Females, including those of Pregnancy and Childbed. By FLEETWOOD CHURCHHILL, M. D., author of "The Theory and Practice of Midwifery," and "Diseases of Infants," &c. A new American edition, revised by the author. With the notes of Robert M. Huston, M. D., Professor of Materia Medica and General Therapeutics in the Jefferson Medical College of Philadelphia, &c. Philadelphia: Lea & Blanchard. 1850. - - - - - - - - - - 524

ART. VII.—Surgical Anatomy. Part 2. By JOSEPH MACLISE, Surgeon. With colored plates. Philadelphia: Lea & Blanchard. 1850. - - - - - - 525

ART. VIII.—A Treatise on Baths; including Cold, Sea, Warm, Vapour, Gas, and Mud Baths; also, of the Watery Regimen, Hydropathy, and Pulmonary Inhalation; with a Description of Bathing in Ancient and Modern Times. By JOHN BELL, M. D., formerly Lecturer on the Institutes of Medicine, and on Materia Medica. Philadelphia: Barrington & Haswell. 1850. - - - - 537

SELECTIONS FROM AMERICAN AND FOREIGN JOURNALS.

Regeneration of the Sciatic nerve, - - - - - - - 531

Pathological Changes that Follow Section of the Sciatic Nerve, 531

Experiments on the Use of Salt, - - - - - - - 532

On the Identity or Non-identity of Typhoid Fever, - - - 535

ORIGINAL INTELLIGENCE.

Demonstrative Midwifery, - - - - - - - 539

An Historical Sketch of the State of Medicine in the American Colonies, - - - - - - - - - 542

Dr. Drake's Treatise, - - - - - - - - - 543

Meeting of the American Medical Association, at Cincinnati, 544

The Ohio Medical and Surgical Journal, - - - - - 544

Dr. Dowler on Death, - - - - - - - - - 545

St. Louis Medical Journal, - - - - - - - - 548

Summer Instruction, - - - - - - - - - 548

